# X-ray Co-crystal Structure of a Novel Pseudomonas aeruginosa DXPS Inhibitor Reveals an Unusual Allosteric Binding Pocket

**DOI:** 10.21203/rs.3.rs-8434964/v1

**Published:** 2026-04-10

**Authors:** Anna Hirsch, Antoine Lacour, Rawia Hamid, Noah Smith, Lydia Krammer, Jörg Haupenthal, Prateek Raj, Sidra Eisa, Dirk Heinz, Mostafa Hamed, Eleonora Diamanti, Caren Freel Meyers

**Affiliations:** Saarland University, HIPS, DZIF; Helmholtz Institute for Pharmaceutical Research Saarland (HIPS); Helmholtz Institute for Pharmaceutical Research Saarland (HIPS); Johns Hopkins University School of Medicine; Helmholtz Institute for Pharmaceutical Research Saarland (HIPS) - Helmholtz Centre for Infection Research (HZI); HZI; Helmholtz Institute for Pharmaceutical Research Saarland (HIPS); Helmholz Centre for Infection Research (HZI); Helmholtz Institute for Pharmaceutical Research Saarland (HIPS); Helmholtz Institute for Pharmaceutical Research Saarland (HIPS); Johns Hopkins School of Medicine

## Abstract

The enzyme 1-deoxy-D-xylulose 5-phosphate synthase (DXPS) catalyzes the first and rate-limiting step of the methylerythritol 4-phosphate (MEP) pathway, representing a promising target for novel anti-infective agents. Given its essential role in the survival of Gram-negative pathogenic bacteria and its absence in humans, drug-discovery efforts to advance our understanding of this enzyme are urgently needed. Here, we unraveled a novel druggable allosteric pocket in DXPS, unexpectedly revealed through co-crystallization of tool compound **14** with *Pseudomonas aeruginosa* DXPS. This inhibitor, identified via virtual screening and subsequent synthetic optimization, binds within an allosteric site distinct from the active site, engaging the protein through halogen bonding interactions. Compound **14** exhibits comparable IC_50_ values against both *P. aeruginosa* and *Klebsiella pneumoniae* DXPS, highlighting its potential as a broad-spectrum DXPS inhibitor. This first co-crystal structure of a non-substrate analog inhibitor with a pathogenic DXPS establishes **14** as a valuable tool compound and provides a novel structural template for future antibiotic development.

## INTRODUCTION

The escalating crisis in public health stemming from infections caused by multidrug-resistant bacteria, is further intensified by the critically limited development of new antibiotics. The current antibiotic-development pipeline is alarmingly narrow, comprising a scant selection of antibacterial agents that offer limited clinical innovation^1,2^. This scenario underscores an urgent need for innovation and investment in antibiotic research and development to address the looming threat posed by pan-drug-resistant bacterial strains.^3^ Over recent decades, the release of novel drugs with unique modes of action has declined due to high development costs and the strategy of reserving new drugs for multidrug-resistant pathogens. Additionally, the World Health Organization’s innovation criteria (requiring new chemical classes, novel mechanisms of action, new targets, and absence of cross-resistance) highlight the urgent need to explore alternative pathways and chemistries.^4^

One promising area of study centers on the methylerythritol 4-phosphate (MEP) pathway, which consists of seven attractive drug targets.^3,5–7^ Named after its second intermediate, this pathway represents one of the two distinct metabolic routes leading to the universal, five-carbon precursors for the biosynthesis of isoprenoids, and is prevalent in most bacteria, plants, and protozoa. In higher eukaryotic organisms and some bacteria, however, the alternative mevalonate pathway is utilized for isoprenoid biosynthesis.^8^

Several pathogens, including *Klebsiella pneumoniae* (*kp*), *Pseudomonas aeruginosa* (*pa*), *Mycobacterium tuberculosis*, and *Plasmodium falciparum*, employ the MEP pathway for the biosynthesis of essential isoprenoids. In contrast, humans rely solely on the mevalonate pathway.^8^ This distinction makes the MEP pathway enzymes promising targets for the development of new anti-infective drugs, owing to the inherent selectivity arising from this metabolic difference.^3^

The enzyme 1-deoxy-D-xylulose 5-phosphate synthase (DXPS) catalyzes the first step of the MEP pathway and is believed to regulate the entire pathway in certain organisms.^9–11^ This first step also represents a branch point in bacterial metabolism as the product 1-deoxy-d-xylulose-5-phosphate (DXP) is additionally involved in vitamin B1 and B6 production.^12,13^ DXPS is a thiamin diphosphate (ThDP)-dependent enzyme that catalyzes the formation of DXP from pyruvate and D-glyceraldehyde 3-phosphate (D-GAP).^14,15^ DXP thus serves as a key intermediate in the biosynthesis of isoprenoid precursors isopentenyl diphosphate (IDP) and dimethylallyl diphosphate (DMADP) as well as vitamins B1 and B6.

DXPS is distinguished from other ThDP-dependent enzymes by several structural and functional properties. A key architectural difference is that its active site is located at the interface between two domains of a single monomer, a departure from enzymes like transketolase (TK) and pyruvate dehydrogenase (PDH), where the active site is located at the dimer interface.^16^ Functionally, DXPS operates via a ligand-gated mechanism requiring ternary complex formation. The catalytic process is also atypical: the enzyme stabilizes the initial C2α-lactyl-ThDP intermediate in the absence of an acceptor substrate, and D-glyceraldehyde 3-phosphate (D-GAP) performs a dual function, first triggering decarboxylation and subsequently acting as the acceptor for DXP production.^14,15,17^

Understanding these unique structural and mechanistic features of DXPS has enabled the development of DXPS-selective inhibitors, qualifying the enzyme as an antibacterial drug target.^18,19^

## RESULTS AND DISCUSSION

### Structure-based virtual screening

Structure-based virtual screening (SBVS) has been widely used for the discovery of novel inhibitors.^20,21^ Herein, we describe our approach to discovering novel inhibitors of DXPS based on crystal structures available from four homologs of the enzyme bound to ThDP. To improve crystallization, three of these homologs were engineered by replacing a flexible loop close to the active site with a short glycine linker: Δ*kp*DXPS (PDB: 8A9C^22^), where 41 residues spanning positions 198–238 were replaced with seven glycine residues; Δ*pa*DXPS (PDB: 8A5K^22^), in which 39 residues from 207–245 were replaced with six glycine residues; and Δ*mt*DXPS (PDB: 7A9H^23^), where 45 residues from 190–234 were replaced with six glycine residues. Using this structural information from the truncated enzymes, alongside the full-length *dr*DXPS (PDB: 2O1X^24^), we screened our in-house library of 3,932 compounds against all four homologs. The goal was to identify a novel class of inhibitors targeting the ThDP binding site while also engaging the substrate binding channel, thereby conferring selectivity for DXPS over other ThDP-dependent enzymes.

To this end, we used SeeSAR^25^ for the initial docking calculations. All poses were then rescored using the recently developed RFScoreVS^26^ scoring function. We then used a combination of KNIME^27^ and Stardrop^28^ to select the most promising potential binders. Compounds that showed highly strained poses or clashes were filtered out. To ensure structural diversity, we clustered the compounds using the ‘Visual Clustering’ method in Stardrop followed by k-means clustering in KNIME^27^, and selected the top hits from each cluster for biological evaluation. Further details on the SBVS protocol and compound selection are available in the Supporting Information. These efforts led to the selection of 266 compounds, of which 140 were available in sufficient quantities for biological evaluation.

[a] 1-deoxy-D-xylulose 5-phosphate synthase [b] Single-point inhibition determined at a compound concentration of 120 μM using the DXPS-IspC coupled activity assay. [b] Means ± standard deviations of at least two independent experiments, using the DXPS LC-MS based assay.

The compounds were initially screened at 120 μM using a DXPS-IspC coupled activity assay on two homologs of DXPS (Δ*pa*DXPS and Δ*kp*DXPS, Δ*mt*DXPS only became available after completion of this screening campaign). The most promising compounds were then evaluated for dose-response using an LC-MS based assay.^29^ This led to the identification of hit **1**, showing promising activity on all the tested DXPS homologs (Table 1). Although the inhibitory potency of **1** was moderate, its modular structure rendered it an attractive starting point for systematic structure–activity relationship (SAR) exploration. We therefore prioritized **1** for further optimization, reasoning that its tractable scaffold would facilitate rapid analog synthesis and enable efficient probing of the DXPS binding site requirements.

### Structure–activity relationships

With the aim of improving the potency of hit **1** and gaining mechanistic insight into its interactions with DXPS, we embarked on a systematic structure–activity relationship (SAR) investigation centered on this chemotype. Our initial focus was to explore the effect of the substituents on the phenyl rings of **1**. The synthesis of these analogs is shown in Scheme 1. Appropriately substituted fluoronitrobenzenes **2a**–**d** were treated with phenols **3a**–**f** under basic conditions to yield nitrophenyl ethers **4a**–**j**. Subsequent iron-mediated reduction of the nitro group yielded substituted phenoxyanilines **5a**–**j**. Amide bond formation with 1-(*tert*-butoxycarbonyl)piperidine-4-carboxylic acid via classical peptide coupling or via the intermediate anhydride afforded amides **6a**–**h**, which were then deprotected to yield free piperidines **7a**–**i**.

i) Cs_2_CO_3_, 50°C, 4 h; ii) Fe, NH_4_Cl_(aq)_, EtOH, 80°C, 3 h; iii) HATU, K_2_CO_3_, 1-(*tert*-butoxycarbonyl)piperidine-4-carboxylic acid, rt, 16 h; iv) NMM, isopropyl chloroformate, 1-(*tert*-butoxycarbonyl)piperidine-4-carboxylic acid, 0°C–rt, 5 h; v) 4 M HCl in dioxane or TFA/DCM, rt, 16 h.

We first aimed to understand the importance of the two chlorine atoms present in hit compound **1** for its activity on the DXPS enzyme. Removal of the terminal chlorine at the R^2^ position (compound **7a**) resulted in a near-total loss of activity against both Δ*pa*DXPS and Δ*kp*DXPS. The complete *des*-chloro analog **7b** was also inactive against either enzyme, confirming the critical role of the halogen substituents for inhibitory activity. Our exploration of the R^1^ position also proved challenging. Replacement of the R^1^ chlorine with a nitrile substituent (compound **7c**), a known isostere for aromatic halogens^30,31^, also led to a complete loss of activity for both enzyme homologs. We next investigated whether the terminal chlorine at R^2^ was involved in a halogen bond. Halogen bonding is characterized by non-covalent interactions involving halogen atoms, driven by the σ-hole — a positively charged region located on the backside of X along the R–X bond axis, resulting from anisotropic distribution of electron density around the R–X bond.^32,33^ To that end, we synthesized a series of halogen-containing compounds and observed a trend consistent with halogen bonding at this position. The fluorine-containing analog (compound **7g**) was significantly less active, showing a 3.9-fold and 8.3-fold loss in activity against Δ*pa*DXPS and Δ*kp*DXPS, respectively, consistent with the inability of fluorine to form halogen bonds. In contrast, substitution with heavier halogens resulted in progressive enhancement of inhibitory potency. The bromo-analog **7h** showed 1.4-fold improvements over compound **1**. The iodo-analog **7i** achieved 2.0- and 1.8-fold improvements against Δ*pa*DXPS and Δ*kp*DXPS, respectively. Exploratory statistical analysis using the Jonckheere–Terpstra trend test^34^ revealed a significant monotonic relationship between halogen identity and inhibitory potency (F < Cl < Br < I; *p* < 0.001 for both enzyme homologs). This correlation is consistent with the established trend of increasing σ-hole character and halogen bond donor strength descending the halogen group.^35^ While these results highlight the importance of the terminal halogen for inhibitor potency, we recognized that further improvements might be achieved through modification of other regions of the scaffold.

DXPS: 1-deoxy-D-xylulose 5-phosphate synthase; inh.: inhibition; N.D.: not determined [a] Single-point inhibition determined at a compound concentration of 120 μM, using DXPS LC-MS based assay. [b] Mean IC_50_ values ± standard deviations of at least two independent experiments, using the DXPS LC-MS based assay.

Encouraged by these results, we then turned our attention to the exploration of the SAR surrounding the piperidinyl ring. To this end, we followed the synthetic route shown in Scheme 2. We synthesized the analogs of **1** with variations around the piperidinyl ring (**8**–**15**) in the same manner as that described in Scheme 1 but using the appropriately substituted acid in the third step. For the synthesis of the reverse amide analog **21**, we instead reacted ester **16** under nucleophilic aromatic substitution conditions with phenol **17** to form ether **18**. Hydrolysis of the ester to acid **19**, followed by amide bond formation to yield **20** and deprotection afforded the appropriately substituted amide **21** (Scheme 2).

i) HATU, K_2_CO_3_, R_1_-CO_2_H, rt, 16 h; ii) optionally: 4 M HCl in dioxane or TFA/DCM, rt, 16 h; iii) Cs_2_CO_3_, 100°C, 72 h; iv) 2 M NaOH, 1,4-dioxane, 50°C, 18 h; v) HATU, K_2_CO_3_, *tert*-butyl 4-(aminomethyl)piperidine-1-carboxylate, rt, 16 h; vi) 4M HCl in dioxane, rt, 16 h.

Subsequently, we systematically investigated the SAR of the piperidinyl ring substitutions and their impact on DXPS inhibitory activity across both enzyme homologs. Analysis of the positioning of the nitrogen atom within the saturated ring system revealed that the 3-N analog (compound **11**) resulted in negligible changes in potency against Δ*pa*DXPS and Δ*kp*DXPS, with differences from compound **1** falling within experimental error. *N*-Methylation of the 4-position nitrogen atom (compound **8**) reduced potency slightly, though the changes (1.4-fold against Δ*pa*DXPS and 1.3-fold against Δ*kp*DXPS) are near the limits of experimental uncertainty. Ring expansion from the 6-membered piperidinyl to the 7-membered azepane (compound **12**) demonstrated divergent effects between the two enzymes, with a 1.5-fold improvement against Δ*kp*DXPS while remaining equipotent on Δ*pa*DXPS. Replacement of the basic nitrogen atom with oxygen (tetrahydropyran, **9**) or elimination of the saturated ring entirely (phenyl, **10**) resulted in complete loss of inhibitory activity against both targets, highlighting the critical importance of the basic nitrogen atom for target engagement. Investigation of linker length between the amide carbonyl and the piperidinyl ring revealed that extension by one methylene unit (compound **13**) afforded promising 2.0-fold and 1.9-fold enhancements in potency against Δ*pa*DXPS and Δ*kp*DXPS, respectively. Further chain extension (compound **14**) provided additional improvement against Δ*pa*DXPS (2.2-fold) and a particularly pronounced 8.6-fold enhancement against Δ*kp*DXPS. Amide bond reversal (compound **21**) resulted in complete loss of inhibitory activity, highlighting the critical importance of the amide directionality. Commercially available analog **15** displayed a significant 1.7-fold decrease in activity against Δ*pa*DXPS while increasing potency against Δ*kp*DXPS (2.0-fold improvement).

DXPS: 1-deoxy-D-xylulose 5-phosphate synthase; N.D.: not determined [a] Mean IC_50_ values ± standard deviations of at least two independent experiments, using the DXPS LC-MS based assay.

### Structural analysis

**Figure 1:**
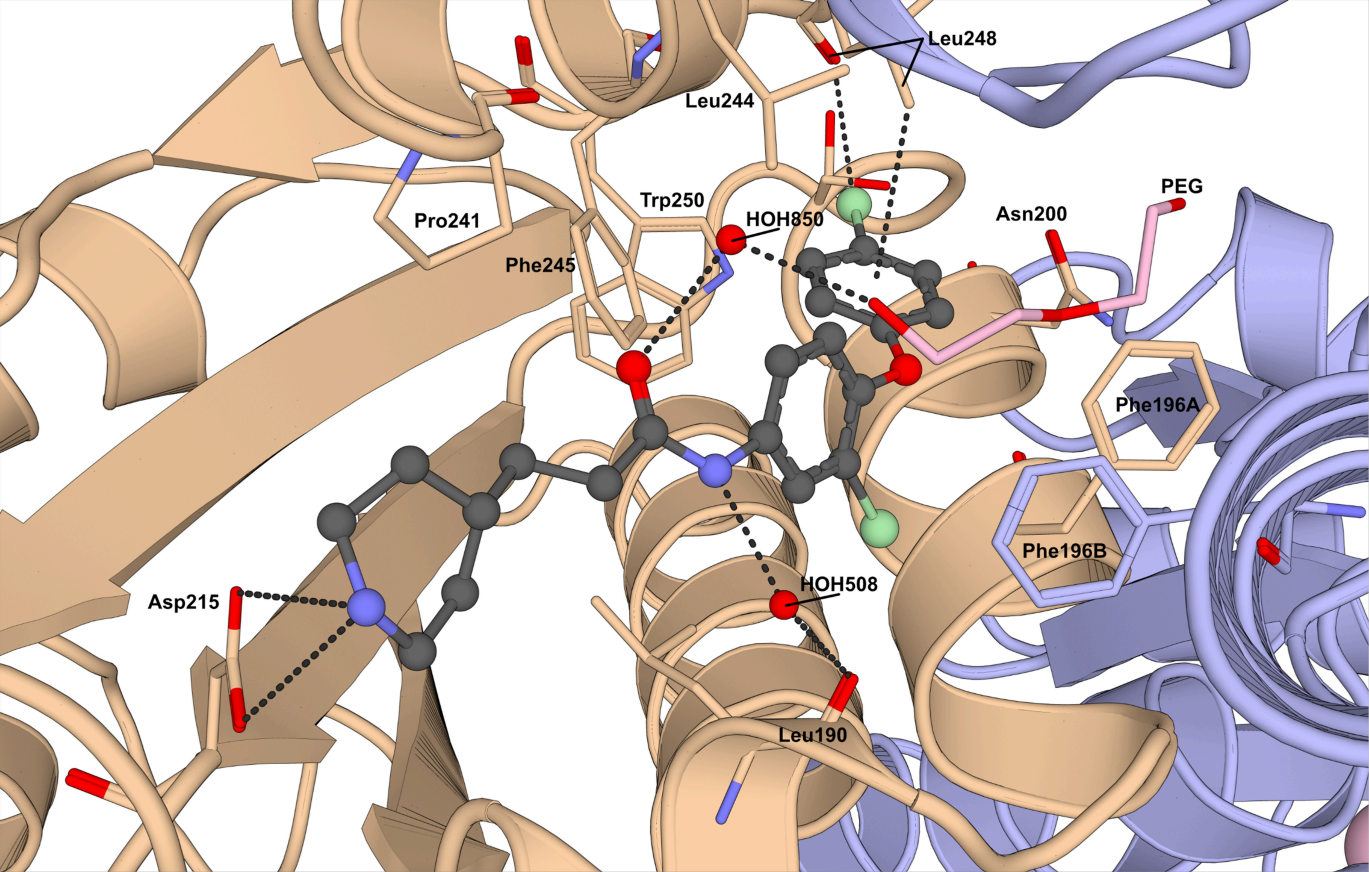
Co-crystal structure of **14**within Δ*pa*DXPS (DXPS: 1-deoxy-D-xylulose 5-phosphate synthase). Inhibitor **14**is shown in dark gray (carbon atoms). Chain A of the Δ*pa*DXPS dimer is shown in beige. Chain B of the Δ*pa*DXPS dimer is shown in light blue. Relevant residues are labeled with a letter depicting the chain where relevant. A PEG molecule present in the crystal is shown in light pink. Important interactions are shown as black, dashed lines. Both solvable positions of Ser203 are shown. Figure generated using PyMol Open Source v3.1.0.4.

**Figure 2:**
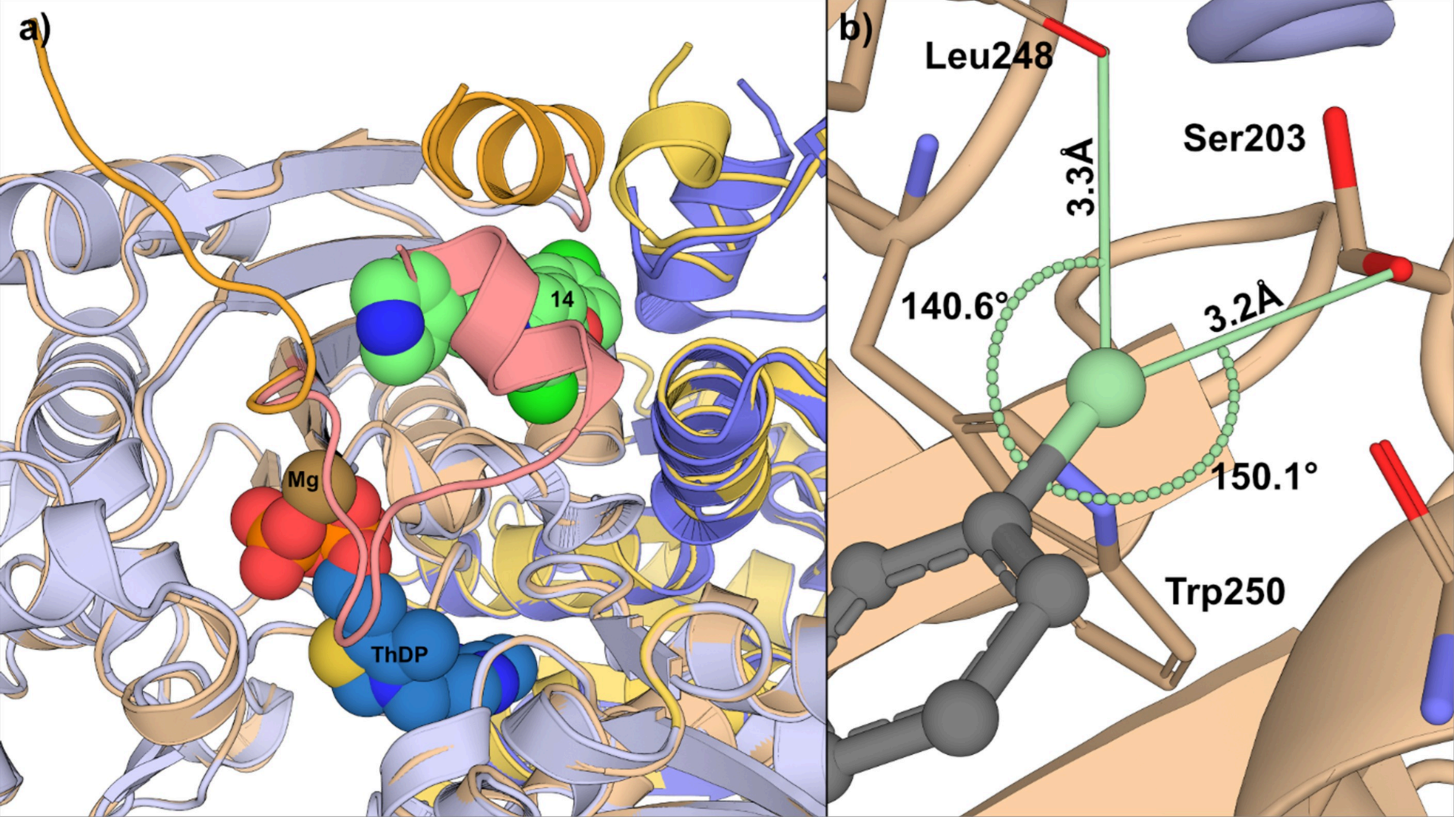
a) Comparison of the thiamine diphosphate (ThDP) (PDB: 8A5K) and compound **14** (PDB: 9QY6) binding site locations. Compound **14** (pale green), ThDP (blue) and the ThDP magnesium atom (brown) are highlighted. The ThDP-bound DXPS backbone is shown in light blue/blue. **14**-bound DXPS backbone is shown in tan/yellow. The flexible loop bearing the truncation is shown in orange (**14**-bound DXPS) and light red (ThDP-bound DXPS). b) Bond angles and distance of potential halogen-bonding interactions with the terminal chlorine (pale green) of compound **14**. Bond angles (in degrees) are shown near the arc defining the angle. Bond distances are expressed in Å. Residue names are shown in bold. Both solvable rotamers of Ser203 are shown.

In order to further understand the mechanism of inhibition of this class of compounds, we proceeded with co-crystallization experiments. We successfully determined the structure of Δ*pa*DXPS bound to compound **14** (PDB: 9QY6, Fig. 1), however, attempts to obtain a complex with Δ*kp*DXPS were unsuccessful. Surprisingly, compound **14** showed an unexpected binding mode, interacting within a small cleft in domain 1 of Δ*pa*DXPS. A previous study from our group showed that this region is involved in significant conformational changes upon ThDP binding (Fig. 2a).^22^ Indeed, compound **14** binds between the flexible loop spanning from Asn216 to Trp250 and a helix formed by residues Asp187 to Glu204. Additionally, compound **14** binds close to the other DXPS monomer and to the site of the truncated loop. The piperidinyl nitrogen atom of compound **14** forms a hydrogen bond with Asp215. This observation corroborates our SAR findings, which showed that compounds lacking a hydrogen-bond donor in this position were inactive (compounds **9** and **10**, Table 3). A bound water molecule (HOH508) is positioned between the amide NH and the backbone carbonyl of Leu190, forming a bridging interaction that connects the ligand to the protein backbone. Both aromatic rings occupy a hydrophobic pocket lined byLeu190, Phe196, Leu199, Leu244, Phe245 and Leu248 (Fig. 1). The terminal aromatic ring engages in a CH–π interaction with the side chain of Leu248, further stabilizing the binding pose. The chlorine atom on the central ring of compound **14** occupies a small, hydrophobic subpocket formed by Leu190, Ala195, Phe196A and Phe196B. The terminal chlorine atom of compound **14** is accommodated at the back of the binding site and is surrounded by Asn200, Ser203, Leu248, Tryp250, Leu199, Gly225B and Gly226B (Fig. 2b). A polyethylene glycol (PEG) molecule is also observed in the binding site; however, this appears to be a crystallization artifact as PEG was used in the crystallization conditions. As our above SAR data suggested the possibility of a halogen bond involving the terminal chlorine in this series of compounds, we analyzed this possibility with regards to the complex structure obtained.

We identified two candidate residues that had the potential to form a halogen bond with the terminal chlorine of compound **14**. The backbone carbonyl of Leu248 forms an angle of 140.6° with respect to the C–Cl bond of compound **14** at an interaction distance of 3.3 Å (Fig. 2b). Ser203 exhibits conformational flexibility, with both rotamers partially resolved in the electron-density data (Fig. 2b). In the rotamer, where the serine hydroxyl group is oriented toward compound **14**, the CCl•••O bond angle is 150.1° at an interaction distance of 3.2 Å. Cl–carbonyl halogen bonds typically exist at a range of angles (140° to 180° with respect to the C–Cl bond) and distances (3 to 5 Å from Cl to O) while typical Cl–serine halogen bonds exist at a distance of 3 to 4 Å and an angle between 130° and 150°.^33^

Based on the structural analysis, we propose that the terminal chlorine atom of compound **14** likely forms a halogen bond with the backbone carbonyl of Leu248. The observed conformational heterogeneity of Ser203, with the halogen bonding-competent rotamer having an occupancy of only 0.35 compared to 0.65 for the alternative conformation, further supports that Ser203 is unlikely to contribute significantly to binding. These structural insights provide a framework for understanding the halogen substitution effects observed in our SAR studies, assuming that compound **14** adopts a similar binding mode to hit compound **1**. These structural insights provide a mechanistic framework for the halogen-substitution effects observed in our SAR studies (F < Cl < Br < I; *p* < 0.001), where the monotonic increase in potency correlates with increasing σ-hole character and halogen-bonding strength. Taken together, these crystallographic and SAR data provide evidence for a halogen-bond interaction between the terminal halogen and the Leu248 backbone carbonyl in compounds **1** and **14**. To further rationalize the observed cross-species inhibitory activity and evaluate whether compound **14** may adopt a similar binding mode in other DXPS orthologs, we next analyzed the sequence conservation of the binding pocket.

### Binding-pocket conservation

To provide a structural rationale for the observed inhibition of both Δ*pa*DXPS and Δ*kp*DXPS, we analyzed the conservation of the compound **14** binding pocket across clinically relevant DXPS orthologs. Residues within 6 Å of compound **14** in the Δ*pa*DXPS co-crystal structure (PDB: 9QY6) were extracted and subjected to multiple sequence alignment with the corresponding regions from *P. aeruginosa* PA14 (UniProt: Q02SL1), *P. aeruginosa* PAO1 (UniProt: Q9KGU7), *K. pneumoniae* 342 (UniProt: B5Y0X1), and *E. coli* K12 (UniProt: P77488).

**Figure 3:**
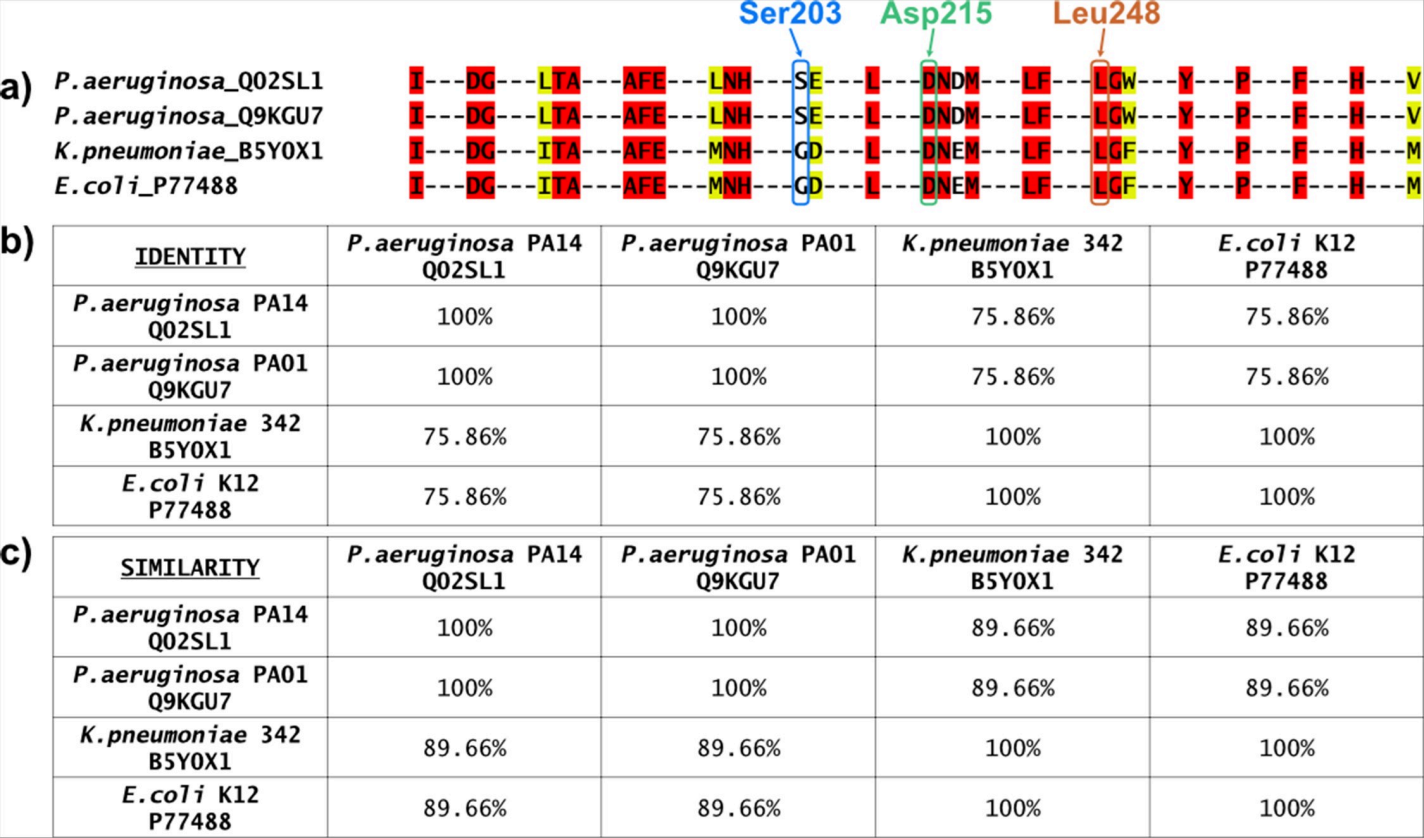
Sequence conservation of the compound **14** binding pocket across 1-deoxy-D-xylulose 5-phosphate synthase (DXPS) orthologs. a): Alignment of residues within 6 Å of compound **14** in Δ*pa*DXPS (PDB: 9QY6). Strictly conserved residues are shown in red; residues with similar physicochemical properties are shown in yellow. Important binding residues are highlighted (Ser203 in blue, Asp215 in green, Leu248 in orange); b): Pairwise sequence identity for the aligned binding-pocket regions; c) Similarity matrix for the aligned binding pocket regions. Sequences were retrieved from UniProt^36^ (*Pseudomonas aeruginosa* PA14, Q02SL1; *P. aeruginosa* PAO1, Q9KGU7; *Klebsiella pneumoniae* 342, B5Y0X1; *Escherichia coli* K12, P77488).

The residues lining the binding pocket exhibited high sequence identity, with 100% identity between the two *P. aeruginosa* strains and 75.86% identity between *P. aeruginosa* and either *K. pneumoniae* or *E. coli* (Figure X). BLOSUM62 similarity scores were correspondingly high, ranging from 0.82 to 0.83 between *P. aeruginosa* and the enterobacterial species, while *K. pneumoniae* and *E. coli* displayed identical binding-pocket sequences (Fig. 3). Notably, key residues involved in ligand recognition are strictly conserved across all four species, including Asp215 (which forms the critical hydrogen bond with the piperidinyl nitrogen atom) and Leu248 (the proposed halogen-bond acceptor, and involved in a CH-π interaction). This high degree of binding-pocket conservation provides a structural basis for the cross-species activity observed in our biochemical assays and suggests that compound **14** likely adopts a similar binding mode in *kp*DXPS. Furthermore, the conservation of the Leu248 backbone carbonyl—identified as the likely halogen-bond acceptor—offers a structural explanation for the consistent halogen substitution SAR (F < < Cl < Br < I) observed across both Δ*pa*DXPS and Δ*kp*DXPS. Ser203 is unique to *P. aeruginosa*, and is replaced by a glycine residue in *K. pneumoniae* and *E. coli*. This suggests that any side chain-mediated halogen bonding to Ser203 would be species-specific, while the interaction with the backbone carbonyl of Leu248 could be maintained across multiple bacterial species. The inclusion of *E. coli* in this analysis, which shares an identical binding pocket with *K. pneumoniae*, prompted us to further investigate whether compound **14** maintains inhibitory activity against *ec*DXPS.

### Inhibition of native DXPS

Given the high binding-pocket conservation between *K. pneumoniae* and *E. coli* DXPS, we sought to confirm that compound **14** also inhibits *ec*DXPS. Furthermore, as initial inhibitor screening and structural analyses were performed using truncated DXPS variants, and given that the crystallographic data revealed the binding site’s proximity to the truncated loop, we aimed to validate that DXPS inhibition by compound **14** was independent of this truncation. We therefore compared the inhibition of compound **14** against full-length *ec*DXPS and Δ*ec*DXPS, in which residues 198–240 are replaced with a hexaglycine linker.

Kinetic characterization of Δ*ec*DXPS for DXP formation (Table 4) revealed comparable *K*_m_ values for pyruvate and d-GAP, and a modest 1.5-fold reduction (*p*-value < 0.0001) in *k*_cat_, relative to WT *ec*DXPS. Compound **14** maintained micromolar inhibitory activity against both enzymes (*K*_i_ = 41.6 and 18.1 μM for full-length and truncated DXPS, respectively) and was determined to be noncompetitive with respect to pyruvate (Table 4). A small, but statistically significant 2.3-fold decrease in the potency of compound **14** was observed on full-length enzyme compared to Δ*ec*DXPS. This difference likely reflects the influence of the mobile loop on the binding site, which is expected given that compound **14** binds in close proximity to the truncation site. Nevertheless, compound **14** maintains micromolar potency on both the WT and truncated enzymes.

## CONCLUSIONS

Our work reports on the identification of a compound with a distinct binding mode targeting an allosteric binding site of the *Pa*DXPS enzyme. Through SBVS and subsequent optimization, we discovered a novel class of diaryl ether amides that demonstrate promising inhibitory activity against DXPS from multiple bacterial species, with an unusual binding mode.

SAR studies highlighted the importance of the terminal halogen and the piperidinyl ring for activity against the DXPS enzyme. The observed trend in activity across different halogen substituents (I > Br > Cl > > F) supported the hypothesis of halogen bonding as a contributing interaction in the binding mode of these compounds. We were able to obtain a co-crystal structure of **14** with Δ*pa*DXPS, revealing an unexpected binding site, involving a small cleft at domain I of the enzyme and halogen bonding to the Leu248 residue. Notably, this represents the first reported co-crystal structure of a non-substrate analog small-molecule inhibitor bound to DXPS from a pathogenic bacterial species. This structure provides a valuable template for structure-based drug design, revealing key interactions including the halogen bond with Leu248 and the hydrogen bond with Asp215, which can guide rational optimization of this scaffold toward more potent and selective DXPS inhibitors. The unique binding mode opens up new avenues for the design of DXPS inhibitors, potentially circumventing selectivity challenges over other ThDP-dependent enzymes relative to ThDP-competitive DXPS inhibitors.

Analysis of binding-pocket conservation revealed high sequence identity (75.9%) and similarity (0.82–0.83) across *P. aeruginosa*, *K. pneumoniae*, and *E. coli* DXPS orthologs, with key binding-site residues strictly conserved. This conservation provides a structural rationale for the observed cross-species inhibitory activity and suggests that this chemotype may possess broad-spectrum potential against Gram-negative pathogens. Compound **14** retained micromolar potency against both full-length and truncated *ec*DXPS, demonstrating that inhibition is not an artifact of the truncation despite the proximity of the binding site to the truncated loop. The 2.3-fold difference in *K*_i_ values between full-length and truncated *ec*DXPS highlights that validation with native enzymes remains essential. Accordingly, truncated variants should be viewed as useful tools for initial screening and structural studies, but not as universal replacements for full-length enzymes in inhibitor development. Additionally, further investigation into the unusual binding mode of these inhibitors may provide insights into the conformational dynamics of DXPS and potentially reveal new strategies for enzyme inhibition.

In conclusion, this study has identified a novel class of DXPS inhibitors as valuable tools for studying DXPS function and serve as promising starting points for the development of new anti-infective agents targeting the MEP pathway. The high-resolution crystal structure of compound **14** in complex with Δ*pa*DXPS provides a precise structural template for the identification of inhibitors that efficiently and selectively target DXPS. The high degree of binding-pocket conservation among DXPS orthologs from clinically relevant Gram-negative pathogens suggests that this scaffold may serve as a useful probe for investigating DXPS enzymes across multiple species. Moreover, the conservation of this allosteric site itself presents an attractive opportunity for the development of broad-spectrum inhibitors capable of targeting DXPS from diverse bacterial pathogens. These findings establish a structural framework for developing more effective DXPS inhibitors to treat infections caused by Gram-negative pathogens, opening a new avenue in the fight against antimicrobial resistance.

## Supplementary Material

Supplementary Files

This is a list of supplementary files associated with this preprint. Click to download.

• V13SINatCommNumbered.docx

• LCMScollectionOptimized.pdf

• NMRcollectionOptimized.pdf

• Table1To3.docx

• Scheme1.png

• Scheme2.png

## Figures and Tables

**Table 4:** *ec*DXPS activity comparison using **14**.

	*ec*DXPS	Δ*ec*DXPS
**K_m_^Pyr^ [μM]** ^[a]^	34.0 ± 3.3	36.6 ± 2.9
**K_m_^D-GAP^ [μM]** ^[a]^	20.4 ± 3.2	26.6 ± 1.8
**k_cat_ [min^−1^]** ^[b]^	41.8 ± 0.8	28.2 ± 1.0
**K_i_^[^μM]** ^[c]^	41.6 ± 2.5	18.1 ± 0.6

DXPS: 1-deoxy-D-xylulose 5-phosphate synthase. The mean and standard error were calculated fromexperiments where n = 4 [a], n = 8 [b], and n = 3 [c].

## References

[1] Miethke M, Pieroni M, Weber T, Brönstrup M, Hammann P, Halby L, Arimondo PB, Glaser P, Aigle B, Bode HB, Moreira R, Li Y, Luzhetskyy A, Medema MH, Pernodet J-L, Stadler M, Tormo JR, Genilloud O, Truman AW, Weissman KJ, Takano E, Sabatini S, Stegmann E, Brötz-Oesterhelt H, Wohlleben W, Seemann M, Empting M, Hirsch AKH, Loretz B, Lehr C-M, Titz A, Herrmann J, Jaeger T, Alt S, Hesterkamp T, Winterhalter M, Schiefer A, Pfarr K, Hoerauf A, Graz H, Graz M, Lindvall M, Ramurthy S, Karlén A, van Dongen M, Petkovic H, Keller A, Peyrane F, Donadio S, Fraisse L, Piddock LJV, Gilbert IH, Moser HE (2021) Müller R. Towards the Sustainable Discovery and Development of New Antibiotics. Nat Rev Chem 5(10):726–749. 10.1038/s41570-021-00313-1 PMC8374425 34426795

[2] Walesch S, Birkelbach J, Jézéquel G, Haeckl FPJ, Hegemann JD, Hesterkamp T, Hirsch AKH, Hammann P, Müller R (2022) Fighting Antibiotic Resistance—Strategies and (Pre)Clinical Developments to Find New Antibacterials. EMBO reports 10.15252/embr.202256033 PMC9827564 36533629

[3] Theuretzbacher U, Blasco B, Duffey M, Piddock LJV (2023) Unrealized Targets in the Discovery of Antibiotics for Gram-Negative Bacterial Infections. Nat Rev Drug Discov 22(12):957–975. 10.1038/s41573-023-00791-6 37833553

[4] Melchiorri D, Rocke T, Alm RA, Cameron AM, Gigante V (2025) Addressing Urgent Priorities in Antibiotic Development: Insights from WHO 2023 Antibacterial Clinical Pipeline Analyses. Lancet Microbe 6(3). 10.1016/j.lanmic.2024.100992 PMC11876093 39454608

[5] Masini T, Kroezen BS, Hirsch AKH (2013) Druggability of the Enzymes of the Non-Mevalonate-Pathway. Drug Discovery Today 18(23–24):1256–1262. 10.1016/j.drudis.2013.07.003 23856326

[6] Wang X, Dowd CS (2018) The Methylerythritol Phosphate Pathway: Promising Drug Targets in the Fight against Tuberculosis. ACS Infect Dis 4(3):278–290. 10.1021/acsinfecdis.7b00176 29390176 PMC6997954

[7] Frank A, Groll M (2017) The Methylerythritol Phosphate Pathway to Isoprenoids. Chem Rev 117(8):5675–5703. 10.1021/acs.chemrev.6b00537 27995802

[8] Heuston S, Begley M, Gahan CGM, Hill C (2012) Isoprenoid Biosynthesis in Bacterial Pathogens. Microbiol (United Kingdom) 158(6):1389–1401. 10.1099/mic.0.051599-0 22466083

[9] Kuzuyama T, Takagi M, Takahashi S, Seto H (2000) Cloning and Characterization of 1-Deoxy-D-Xylulose 5-Phosphate Synthase from Streptomyces Sp. Strain CL190, Which Uses Both the Mevalonate and Nonmevalonate Pathways for Isopentenyl Diphosphate Biosynthesis. J Bacteriol 182(4):891–897. 10.1128/JB.182.4.891-897.2000 10648511 PMC94361

[10] Estévez JM, Cantero A, Reindl A, Reichler S, León P (2001) 1-Deoxy-D-Xylulose-5-Phosphate Synthase, a Limiting Enzyme for Plastidic Isoprenoid Biosynthesis in Plants. J Biol Chem 276(25):22901–22909. 10.1074/jbc.M100854200 11264287

[11] Gierse RM, Redeem E, Diamanti E, Wrenger C, Groves MR, Hirsch AK Dxs as a Target for Structure-Based Drug Design. Future Med Chem 2017, 9 (11), 1277–1294. 10.4155/fmc-2016-0239 28636418

[12] Himmeldirk K, Kennedy IA, Hill RE, Sayer BG, Spenser ID (1996) Biosynthesis of Vitamins B1 and B6 in Escherichia Coli: Concurrent Incorporation of 1-Deoxy-D-Xylulose into Thiamin (B1) and Pyridoxol (B6). Chem Commun 101187–1188. 10.1039/CC9960001187

[13] Hill RE, Himmeldirk K, Kennedy IA, Pauloski RM, Sayer BG, Wolf E, Spenser ID (1996) The Biogenetic Anatomy of Vitamin B6: A 13C NMR Investigation of the Biosynthesis of Pyridoxol in Escherichia Coli. J Biol Chem 271(48):30426–30435. 10.1074/jbc.271.48.30426 8940007

[14] Brammer LA, Smith JM, Wades H, Meyers CF (2011) 1-Deoxy-D-Xylulose 5-Phosphate Synthase Catalyzes a Novel Random Sequential Mechanism. J Biol Chem 286(42):36522–36531. 10.1074/jbc.M111.259747 21878632 PMC3196101

[15] Brammer Basta LA, Patel H, Kakalis L, Jordan F, Freel Meyers CL (2014) Defining Critical Residues for Substrate Binding to 1-Deoxy-d-Xylulose 5-Phosphate Synthase - Active Site Substitutions Stabilize the Predecarboxylation Intermediate C2α-Lactylthiamin Diphosphate. FEBS J 281(12):2820–2837. 10.1111/febs.12823 24767541 PMC4065394

[16] Bartee D, Freel Meyers CL (2018) Toward Understanding the Chemistry and Biology of 1-Deoxy-d-Xylulose 5-Phosphate (DXP) Synthase: A Unique Antimicrobial Target at the Heart of Bacterial Metabolism. Acc Chem Res 51(10):2546–2555. 10.1021/acs.accounts.8b00321 30203647 PMC6309272

[17] Smith JM, Warrington NV, Vierling RJ, Kuhn ML, Anderson WF, Koppisch AT, Freel Meyers CL (2014) Targeting DXP Synthase in Human Pathogens: Enzyme Inhibition and Antimicrobial Activity of Butylacetylphosphonate. J Antibiot 67(1):77–83. 10.1038/ja.2013.105 PMC3946878 24169798

[18] Bartee D, Freel Meyers CL (2018) Targeting the Unique Mechanism of Bacterial 1-Deoxy-d-Xylulose-5-Phosphate Synthase. Biochemistry 57(29):4349–4356. 10.1021/acs.biochem.8b00548 29944345 PMC6057799

[19] Masini T, Hirsch AKH (2014) Development of Inhibitors of the 2C-Methyl-D-Erythritol 4-Phosphate (MEP) Pathway Enzymes as Potential Anti-Infective Agents. J Med Chem 57(23):9740–9763. 10.1021/jm5010978 25210872

[20] Lionta E, Spyrou G, Vassilatis D, Cournia Z (2014) Structure-Based Virtual Screening for Drug Discovery: Principles, Applications and Recent Advances. CTMC 14 (16), 1923–1938. 10.2174/1568026614666140929124445 PMC4443793 25262799

[21] Danishuddin M, Khan AU (2015) Structure Based Virtual Screening to Discover Putative Drug Candidates: Necessary Considerations and Successful Case Studies. Methods 71, 135–145. 10.1016/j.ymeth.2014.10.019 25448480

[22] Hamid R, Adam S, Lacour A, Monjas L, Köhnke J, Hirsch AKH (2023) 1-Deoxy-D-Xylulose-5-Phosphate Synthase from Pseudomonas Aeruginosa and Klebsiella Pneumoniae Reveals Conformational Changes upon Cofactor Binding. J Biol Chem 299(9):105152. 10.1016/j.jbc.2023.105152 37567475 PMC10504544

[23] Gierse RM, Oerlemans R, Reddem ER, Gawriljuk VO, Alhayek A, Baitinger D, Jakobi H, Laber B, Lange G, Hirsch AKH, Groves MR (2022) First Crystal Structures of 1-Deoxy-D-Xylulose 5-Phosphate Synthase (DXPS) from Mycobacterium Tuberculosis Indicate a Distinct Mechanism of Intermediate Stabilization. Sci Rep 12(1):7221. 10.1038/s41598-022-11205-9 35508530 PMC9068908

[24] Xiang S, Usunow G, Lange G, Busch M, Tong L (2007) Crystal Structure of 1-Deoxy-D-Xylulose 5-Phosphate Synthase, a Crucial Enzyme for Isoprenoids Biosynthesis. J Biol Chem 282(4):2676–2682. 10.1074/jbc.M610235200 17135236

[25] BioSolveIT GmbH SeeSAR Version 10.3, 2021.

[26] Wójcikowski M, Ballester PJ, Siedlecki P (2017) Performance of Machine-Learning Scoring Functions in Structure-Based Virtual Screening. Sci Rep 7(1):1–10. 10.1038/srep46710 28440302 PMC5404222

[27] Berthold MR, Cebron N, Dill F, Gabriel TR, Kötter T, Meinl T, Ohl P, Sieb C, Thiel K, Wiswedel BKNIME(2008) The Konstanz Information Miner; Springer,; pp 319–326. 10.1007/978-3-540-78246-9_38

[28] Optibrium Stardrop (2021) https://optibrium.com/stardrop/ (accessed 2024-01-03).

[29] Liang Y-F, Liu H, Li H, Gao W-Y (2019) Determination of the Activity of 1-Deoxy-D-Xylulose 5-Phosphate Synthase by Pre-Column Derivatization-HPLC Using 1,2-Diamino-4,5-Methylenedioxybenzene as a Derivatizing Reagent. Protein J 38(2):160–166. 10.1007/s10930-019-09816-9 30707333

[30] Fleming FF, Yao L, Ravikumar PC, Funk L, Shook BC (2010) Nitrile-Containing Pharmaceuticals: Efficacious Roles of the Nitrile Pharmacophore. J Med Chem 53(22):7902–7917. 10.1021/jm100762r 20804202 PMC2988972

[31] Leeson PD, Springthorpe B (2007) The Influence of Drug-like Concepts on Decision-Making in Medicinal Chemistry. Nat Rev Drug Discovery 6(11):881–890. 10.1038/nrd2445 17971784

[32] Clark T, Hennemann M, Murray JS, Politzer PH, Bonding (2007) The σ-Hole: Proceedings of Modeling Interactions in Biomolecules II, Prague, September 5th–9th, 2005. J Mol Model 13 (2), 291–296. 10.1007/s00894-006-0130-2 16927107

[33] Wilcken R, Zimmermann MO, Lange A, Joerger AC, Boeckler FM (2013) Principles and Applications of Halogen Bonding in Medicinal Chemistry and Chemical Biology. J Med Chem 56(4):1363–1388. 10.1021/jm3012068 23145854

[34] Jonckheere AR (1954) A Distribution-Free k-Sample Test Against Ordered Alternatives. Biometrika 41(1/2):133–145. 10.2307/2333011

[35] Cavallo G, Metrangolo P, Milani R, Pilati T, Priimagi A, Resnati G, Terraneo G (2016) The Halogen Bond. Chem Rev 116(4):2478–2601. 10.1021/acs.chemrev.5b00484 26812185 PMC4768247

[36] Bateman A, Martin M-J, Orchard S, Magrane M, Ahmad S, Alpi E, Bowler-Barnett EH, Britto R, Bye-A-Jee H, Cukura A, Denny P, Dogan T, Ebenezer T, Fan J, Garmiri P, da Costa Gonzales LJ, Hatton-Ellis E, Hussein A, Ignatchenko A, Insana G, Ishtiaq R, Joshi V, Jyothi D, Kandasaamy S, Lock A, Luciani A, Lugaric M, Luo J, Lussi Y, MacDougall A, Madeira F, Mahmoudy M, Mishra A, Moulang K, Nightingale A, Pundir S, Qi G, Raj S, Raposo P, Rice DL, Saidi R, Santos R, Speretta E, Stephenson J, Totoo P, Turner E, Tyagi N, Vasudev P, Warner K, Watkins X, Zaru R, Zellner H, Bridge AJ, Aimo L, Argoud-Puy G, Auchincloss AH, Axelsen KB, Bansal P, Baratin D, Batista Neto TM, Blatter M-C, Bolleman JT, Boutet E, Breuza L, Gil BC, Casals-Casas C, Echioukh KC, Coudert E, Cuche B, de Castro E, Estreicher A, Famiglietti ML, Feuermann M, Gasteiger E, Gaudet P, Gehant S, Gerritsen V, Gos A, Gruaz N, Hulo C, Hyka-Nouspikel N, Jungo F, Kerhornou A, Le Mercier P, Lieberherr D, Masson P, Morgat A, Muthukrishnan V, Paesano S, Pedruzzi I, Pilbout S, Pourcel L, Poux S, Pozzato M, Pruess M, Redaschi N, Rivoire C, Sigrist CJA, Sonesson K, Sundaram S, Wu CH, Arighi CN, Arminski L, Chen C, Chen Y, Huang H, Laiho K, McGarvey P, Natale DA, Ross K, Vinayaka Q, Wang Y, Zhang (2023) J. UniProt: The Universal Protein Knowledgebase in 2023. Nucleic Acids Research 51 (D1), D523–D531. 10.1093/nar/gkac1052 36408920 PMC9825514

